# Supplementary material for: Fast tensorial JADE
Source: Scand Stat Theory Appl. 2020 Feb 11;48(1):164–87. doi: 10.1111/sjos.12445 (PMC7891388; doi:10.1111/sjos.12445)
Supplement: Supplementary file 1 — Data S1: Supporting Information. [file SJOS-48-164-s001.zip › SJOS12445-sup-0001-supinfo.pdf]

Appendix to “Fast tensorial JADE”

Joni Virta[1,2] | Niko Lietzén[1] | Pauliina Ilmonen[1] | Klaus Nordhausen[3]

[1]Department of Mathematics and Systems Analysis, Aalto University School of Science, Finland

[2]Department of Mathematics and Statistics, University of Turku, Finland

[3]Institute of Statistics & Mathematical Methods in Economics, Vienna University of Technology, Austria

1 | PROOFS

We denote the sequence of i.i.d. observations as $\mathbf{X}_N = \{\mathbf{X}_1, \ldots, \mathbf{X}_n\}$, such that e.g. $\hat{\boldsymbol{\Sigma}}_1[\mathbf{X}_N]$ denotes the left sample covariance matrix estimated from the sample $\mathbf{X}_N$.
Before the proofs of the main results, we establish three auxiliary lemmas.

Lemma 1 *The minimization problem* $\mathrm{argmin}_{\mathbf{V} \in \mathcal{U}} \{\tilde{g}(\mathbf{V}, \mathbf{X})\}$ *is equivalent to the maximization problem* $\mathrm{argmax}_{\mathbf{V} \in \mathcal{U}} \{g(\mathbf{V}, \mathbf{X})\}$, *where* $\mathcal{U} = \{\mathbf{V} \in \mathcal{R}^{p \times p} : \mathbf{V}\mathbf{V}' = \mathbf{I}_p\}$,

$$\tilde{g}(\mathbf{V}, \mathbf{X}) = \sum_{i,j=1}^{p} \left\| \mathrm{off}\left(\mathbf{V}'\mathbf{C}^{ij}[\mathbf{X}]\mathbf{V}\right) \right\|_F^2 \quad \text{and} \quad g(\mathbf{V}, \mathbf{X}) = \sum_{i,j=1}^{p} \left\| \mathrm{diag}\left(\mathbf{V}'\mathbf{C}^{ij}[\mathbf{X}]\mathbf{V}\right) \right\|_F^2 .$$

Proof of Lemma 1 Note that for all $\mathbf{V} \in \mathcal{U}$,

$$\left\| \mathbf{C}^{ij}[\mathbf{X}] \right\|_F^2 = \left\| \mathbf{V}'\mathbf{C}^{ij}[\mathbf{X}]\mathbf{V} \right\|_F^2 = \left\| \mathrm{diag}\left(\mathbf{V}'\mathbf{C}^{ij}[\mathbf{X}]\mathbf{V}\right) \right\|_F^2 + \left\| \mathrm{off}\left(\mathbf{V}'\mathbf{C}^{ij}[\mathbf{X}]\mathbf{V}\right) \right\|_F^2 .$$

Thus, $\underset{\mathbf{V} \in \mathcal{U}}{\mathrm{argmin}} \{\tilde{g}(\mathbf{V}, \mathbf{X})\}$ is equivalent to

$$\underset{\mathbf{V} \in \mathcal{U}}{\mathrm{argmax}} \left\{ \sum_{i,j=1}^{p} \left(\left\| \mathrm{diag}\left(\mathbf{V}'\mathbf{C}^{ij}[\mathbf{X}]\mathbf{V}\right) \right\|_F^2 - \left\| \mathbf{C}^{ij}[\mathbf{X}] \right\|_F^2 \right) \right\} = \underset{\mathbf{V} \in \mathcal{U}}{\mathrm{argmax}} \{g(\mathbf{V}, \mathbf{X})\} .$$

Lemma 2 *For all $(i, j) \in \{1, \dots, p\} \times \{1, \dots, p\}$ and any orthogonal matrix $\boldsymbol{H} = (h_{ij}) \in \mathcal{R}^{p \times p}$, the sample and the population cumulant matrices satisfy,*

$$\boldsymbol{H}'\boldsymbol{C}^{ij}[\boldsymbol{HX}]\boldsymbol{H} = \sum_{a=1}^{p}\sum_{b=1}^{p} h_{ia}h_{jb}\boldsymbol{C}^{ab}[\boldsymbol{X}] \tag{8}$$
$$\boldsymbol{H}'\hat{\boldsymbol{C}}^{ij}[\boldsymbol{HX}_N]\boldsymbol{H} = \sum_{a=1}^{p}\sum_{b=1}^{p} h_{ia}h_{jb}\hat{\boldsymbol{C}}^{ab}[\boldsymbol{X}_N],$$

where $\boldsymbol{HX}_N$ denotes the sample $\{\boldsymbol{HX}_1, \dots, \boldsymbol{HX}_n\}$.

Proof of Lemma 2 Both results follow by the same arguments and we prove only the former. Since $\mathbf{e}_i'\mathbf{XX}'\mathbf{e}_j$ is a scalar and $\boldsymbol{\Sigma}_1[\mathbf{HX}] = \mathbf{H}\boldsymbol{\Sigma}_1[\mathbf{X}]\mathbf{H}'$, the left-hand side of Eq. (8) can be written as

$$\frac{1}{q}\mathbb{E}[\mathbf{e}_i'\mathbf{HXX}'\mathbf{H}'\mathbf{e}_j\mathbf{XX}'] - \boldsymbol{\Sigma}_1[\mathbf{X}]\mathbf{H}'(\delta_{ij}q\mathbf{I}_p + \mathbf{E}^{ij} + \mathbf{E}^{ji})\mathbf{H}(\boldsymbol{\Sigma}_1[\mathbf{X}])', \tag{9}$$

where the first term can be written as

$$\sum_{a,b}\frac{1}{q}\mathbb{E}[h_{ia}(\mathbf{XX}')_{ab}h_{jb}\mathbf{XX}'] = \sum_{a,b} h_{ia}h_{jb}\frac{1}{q}\mathbb{E}[\mathbf{e}_a'\mathbf{XX}'\mathbf{e}_b\mathbf{XX}'], \tag{10}$$

where the $1/q$-scaled expected value is the first term in the definition of the matrix $\mathbf{C}^{ab}[\mathbf{X}]$ and has the representation

$$\frac{1}{q}\mathbb{E}[\mathbf{e}_a'\mathbf{XX}'\mathbf{e}_b\mathbf{XX}'] = \mathbf{C}^{ab}[\mathbf{X}] + \boldsymbol{\Sigma}_1[\mathbf{X}](\delta_{ab}q\mathbf{I}_p + \mathbf{E}^{ab} + \mathbf{E}^{ba})(\boldsymbol{\Sigma}_1[\mathbf{X}])'. \tag{11}$$

Plugging Eq. (11) into Eq. (10) we obtain

$$\begin{aligned}&\sum_{a,b}\frac{1}{q}\mathbb{E}[h_{ia}(\mathbf{XX}')_{ab}h_{jb}\mathbf{XX}'] \\ &= \sum_{a,b} h_{ia}h_{jb}\left(\mathbf{C}^{ab}[\mathbf{X}] + \boldsymbol{\Sigma}_1[\mathbf{X}](\delta_{ab}q\mathbf{I}_p + \mathbf{E}^{ab} + \mathbf{E}^{ba})(\boldsymbol{\Sigma}_1[\mathbf{X}])'\right).\end{aligned} \tag{12}$$

Furthermore, plugging Eq. (12) into Eq. (9) gives us,

$$\begin{aligned}\mathbf{H}'\mathbf{C}^{ij}[\mathbf{HX}]\mathbf{H} = &\sum_{a,b} h_{ia}h_{jb}\left(\mathbf{C}^{ab}[\mathbf{X}] + \boldsymbol{\Sigma}_1[\mathbf{X}](\delta_{ab}q\mathbf{I}_p + \mathbf{E}^{ab} + \mathbf{E}^{ba})(\boldsymbol{\Sigma}_1[\mathbf{X}])'\right) \\ &- \boldsymbol{\Sigma}_1[\mathbf{X}]\mathbf{H}'(\delta_{ij}q\mathbf{I}_p + \mathbf{E}^{ij} + \mathbf{E}^{ji})\mathbf{H}(\boldsymbol{\Sigma}_1[\mathbf{X}])'.\end{aligned} \tag{13}$$

Element-wise examination reveals that,

$$\sum_{a,b} h_{ia}h_{jb}\boldsymbol{\Sigma}_1[\mathbf{X}](\delta_{ab}q\mathbf{I}_p)(\boldsymbol{\Sigma}_1[\mathbf{X}])' - \boldsymbol{\Sigma}_1[\mathbf{X}]\mathbf{H}'(\delta_{ij}q\mathbf{I}_p)\mathbf{H}(\boldsymbol{\Sigma}_1[\mathbf{X}])' = \mathbf{0},$$
$$\sum_{a,b} h_{ia}h_{jb}\boldsymbol{\Sigma}_1[\mathbf{X}]\mathbf{E}^{ab}(\boldsymbol{\Sigma}_1[\mathbf{X}])' - \boldsymbol{\Sigma}_1[\mathbf{X}]\mathbf{H}'\mathbf{E}^{ij}\mathbf{H}(\boldsymbol{\Sigma}_1[\mathbf{X}])' = \mathbf{0}, \quad \text{and}$$
$$\sum_{a,b} h_{ia}h_{jb}\boldsymbol{\Sigma}_1[\mathbf{X}]\mathbf{E}^{ba}(\boldsymbol{\Sigma}_1[\mathbf{X}])' - \boldsymbol{\Sigma}_1[\mathbf{X}]\mathbf{H}'\mathbf{E}^{ji}\mathbf{H}(\boldsymbol{\Sigma}_1[\mathbf{X}])' = \mathbf{0}.$$

This concludes the proof.

Lemma 3 *Let* $\hat{\mathbf{S}} \in \mathcal{R}^{p\times p}$ *be a sequence of estimators (indexed by* n*) such that* $\sqrt{n}(\hat{\mathbf{S}} - \mathbf{\Lambda}) \rightsquigarrow \mathcal{D}$*, where* $\rightsquigarrow$ *denotes convergence in distribution,* $\mathcal{D}$ *is some matrix-valued distribution and*

$$\mathbf{\Lambda} = \begin{pmatrix} \lambda_1 \mathbf{I}_{k_1} & \mathbf{0} & \cdots & \mathbf{0} \\ \mathbf{0} & \lambda_2 \mathbf{I}_{k_2} & \cdots & \mathbf{0} \\ \vdots & \vdots & \ddots & \vdots \\ \mathbf{0} & \mathbf{0} & \cdots & \lambda_R \mathbf{I}_{k_R} \end{pmatrix},$$

where $k_1 + \cdots + k_R = p$ *and the values* λ_k, $k \in \{1, \ldots, R\}$*, are distinct and in a strictly decreasing order* ($\lambda_1 > \lambda_2 > \cdots > \lambda_R$)*. Let* $\hat{\mathbf{U}}$ *be the sequence of eigenvector matrices, where the columns are the eigenvectors of the matrices* $\hat{\mathbf{S}}$*. Partition* $\hat{\mathbf{U}}$ *into blocks* $\hat{\mathbf{U}}_{ij} \in \mathcal{R}^{k_i \times k_j}$ *as*

$$\hat{\mathbf{U}} = \begin{pmatrix} \hat{\mathbf{U}}_{11} & \hat{\mathbf{U}}_{12} & \cdots & \hat{\mathbf{U}}_{1R} \\ \hat{\mathbf{U}}_{21} & \hat{\mathbf{U}}_{22} & \cdots & \hat{\mathbf{U}}_{2R} \\ \vdots & \vdots & \ddots & \vdots \\ \hat{\mathbf{U}}_{R1} & \hat{\mathbf{U}}_{R2} & \cdots & \hat{\mathbf{U}}_{RR} \end{pmatrix},$$

in a similar way to $\mathbf{\Lambda}$*. Then*

$$\hat{\mathbf{U}}_{ij} = O_p\left(\frac{1}{\sqrt{n}}\right), \quad \textit{whenever} \quad i \neq j.$$

Proof of Lemma 3 The sequence of eigenvectors satisfies the eigenequation

$$\hat{\mathbf{S}}\hat{\mathbf{U}} = \hat{\mathbf{U}}\hat{\mathbf{\Lambda}}, \tag{14}$$

where $\hat{\mathbf{\Lambda}}$ is a sequence of diagonal matrices containing the estimated eigenvalues. Then,

$$\sqrt{n}\hat{\mathbf{S}}\hat{\mathbf{U}} = \sqrt{n}(\hat{\mathbf{S}} - \mathbf{\Lambda})\hat{\mathbf{U}} + \sqrt{n}\mathbf{\Lambda}\hat{\mathbf{U}} = \sqrt{n}\mathbf{\Lambda}\hat{\mathbf{U}} + O_p(1), \tag{15}$$

where the second equality holds since $\hat{\mathbf{U}} = O_p(1)$ as a result of the compactness of the space of orthogonal matrices and since, by Prohorov's theorem, our assumption $\sqrt{n}(\hat{\mathbf{S}} - \mathbf{\Lambda}) \rightsquigarrow \mathcal{D}$ implies that $\sqrt{n}(\hat{\mathbf{S}} - \mathbf{\Lambda}) = O_p(1)$.

It follows from (Eaton and Tyler, 1991, Theorem 3.2) that $\sqrt{n}(\hat{\mathbf{\Lambda}} - \mathbf{\Lambda}) = O_p(1)$, and consequently we obtain

$$\sqrt{n}\hat{\mathbf{U}}\hat{\mathbf{\Lambda}} = \hat{\mathbf{U}}\sqrt{n}(\hat{\mathbf{\Lambda}} - \mathbf{\Lambda}) + \sqrt{n}\hat{\mathbf{U}}\mathbf{\Lambda} = \sqrt{n}\hat{\mathbf{U}}\mathbf{\Lambda} + O_p(1), \tag{16}$$

By combining Eqs. (15) and (16) with Eq. (14), we arrive at

$$\mathbf{\Lambda}\hat{\mathbf{U}} - \hat{\mathbf{U}}\mathbf{\Lambda} = O_p\left(\frac{1}{\sqrt{n}}\right).$$

If this is written block-wise, we obtain

$$(\lambda_i - \lambda_j)\hat{\mathbf{U}}_{ij} = O_p\left(\frac{1}{\sqrt{n}}\right), \quad \text{for all} \quad (i,j) \in \{1,\dots,R\} \times \{1,\dots,R\}.$$

The result now follows by considering only the off-diagonal blocks, $i \neq j$, and dividing by the non-zero $(\lambda_i - \lambda_j)$.

Remark In the sequel, the assumption of Lemma 3 that $\sqrt{n}(\hat{\mathbf{S}} - \mathbf{\Lambda}) \rightsquigarrow \mathcal{D}$ is fulfilled by applying central limit theorem.

Proof of Theorem 1 Let Assumptions 1 and 2(v) hold for some fixed v. Consider now the k-TJADE functional $\hat{\mathbf{\Gamma}}^k$ where $k \geq v$.

By (Virta et al., 2017, Theorem 1) the standardized matrix satisfies $\mathbf{X}^{\text{st}} = \tau \mathbf{U}_1 \mathbf{Z} \mathbf{U}_2'$ for some $\tau > 0$ and for some orthogonal matrices, $\mathbf{U}_1 \in \mathcal{R}^{p \times p}, \mathbf{U}_2 \in \mathcal{R}^{q \times q}$. By (Virta et al., 2017, Eq. (5)) the TFOBI-matrix functional $\mathbf{B}[\mathbf{X}^{\text{st}}]$ has the form,

$$\mathbf{B}[\mathbf{X}^{\text{st}}] = \mathbf{U}_1 \left(\sum_{k=1}^{p} \tau^4 \left(\kappa_k + p + q + 1\right) \mathbf{E}^{kk} \right) \mathbf{U}_1',$$

where, $\kappa_k = (1/q)\sum_{j=1}^{q} \mathbb{E}[z_{kj}^4] - 3$, are the row means of the kurtosis values of $\mathbf{Z} = (z_{ij})$ such that $\kappa_1 \geq \cdots \geq \kappa_p$. Let R be the number of the distinct eigenvalues of $\mathbf{B}[\mathbf{X}^{\text{st}}]$. Denote these distinct values by $\lambda_1 > \cdots > \lambda_R$ and denote the corresponding multiplicities of these values by $k_1,\dots,k_R$, respectively. Note that each λ_r can be given as $\tau^4\left(\kappa_k + p + q + 1\right)$ for some k. As $k \geq v$, where v is the largest multiplicity among the row mean kurtoses $\kappa_1,\dots,\kappa_p$, we have that $k_r \leq k$, for all r.

The set of eigenvectors of $\mathbf{B}[\mathbf{X}^{\text{st}}]$ is identifiable up to orthogonal transformations within each eigenspace. That is, ignoring the order and signs, the eigenvector matrix $\mathbf{W}_1$ of $\mathbf{B}[\mathbf{X}^{\text{st}}]$ has the form

$$\mathbf{W}_1 = \mathbf{U}_1 \begin{pmatrix} \mathbf{H}_{11}' & \mathbf{0} & \cdots & \mathbf{0} \\ \mathbf{0} & \mathbf{H}_{12}' & \cdots & \mathbf{0} \\ \vdots & \vdots & \ddots & \vdots \\ \mathbf{0} & \mathbf{0} & \cdots & \mathbf{H}_{1R}' \end{pmatrix} = \mathbf{U}_1 \mathbf{H}_1', \tag{17}$$

where each $\mathbf{H}_{1r} \in \mathcal{R}^{k_r \times k_r}$, $r \in \{1,\dots,R\}$, is orthogonal. The FOBI-rotated data is then

$$\mathbf{X}^{\text{F}} = \mathbf{\Gamma}^{\text{F}}[\mathbf{X}]\mathbf{X}(\mathbf{\Gamma}^{\text{F}}[\mathbf{X}'])' = \mathbf{W}_1'\mathbf{X}^{\text{st}}\mathbf{W}_2 = \tau \mathbf{H}_1 \mathbf{Z} \mathbf{H}_2'.$$

The proof of Theorem 1 is divided into two parts. First, we prove that the condition *(i)* in Definition 1 holds and after that we prove that the condition *(ii)* holds.

Condition *(i)*:

The condition *(i)* claims that k-TJADE can, under Assumptions 1 and 2(v), estimate the block diagonal orthogonal matrix $\mathbf{H}_1$ up to the signs and the order of its columns. For convenience, we drop all subscripts referring to the side (left or right) of the model, e.g. in the following $\mathbf{H}_{1r}$ is $\mathbf{H}_r$ and $\mathbf{H}_1$ is $\mathbf{H}$.

Adapting the proof of (Virta et al., 2018, Theorem 1), we have that

$$\mathbf{C}^{ij}[\mathbf{X}^{\text{F}}] = \mathbf{H}\left(\sum_{k=1}^{p} \tau^4 h_{ik} h_{jk} \kappa_k \mathbf{E}^{kk} \right) \mathbf{H}' = \mathbf{H}\mathbf{D}^{ij}\mathbf{H}', \tag{18}$$

where $\mathbf{D}^{ij} = \sum_{k=1}^{p} \tau^4 h_{ik} h_{jk} \kappa_k \mathbf{E}^{kk}$ is diagonal matrix for every $i, j \in \{1, \ldots, p\}$. Since $\tilde{g}(\mathbf{H}, \mathbf{X}^{\mathsf{F}}) = 0$, the matrix $\mathbf{H}$ is a solution to the k-TJADE minimization problem

$$\min_{\{\mathbf{V}:\mathbf{V}\mathbf{V}'=\mathbf{I}_p\}} \left\{ \tilde{g}(\mathbf{V}, \mathbf{X}^{\mathsf{F}}) \right\} = \min_{\{\mathbf{V}:\mathbf{V}\mathbf{V}'=\mathbf{I}_p\}} \left\{ \sum_{|i-j|<k} \left\| \mathrm{off} \left(\mathbf{V}' \mathbf{C}^{ij} [\mathbf{X}^{\mathsf{F}}] \mathbf{V} \right) \right\|_{\mathsf{F}}^2 \right\}.$$

Next, we show that $\mathbf{H}$ is a unique minimizer, up to the signs and the order of its columns. Let $K_r = \sum_{m=1}^{r} k_r$ such that $K_0 = 0$ and let $\mathcal{I}_r = \{K_{r-1} + 1, K_{r-1} + 2, \ldots, K_r\}$ be the subset of the index set $\{1, \ldots, p\}$ for which the corresponding columns of $\mathbf{H}$ belong to the rth eigenvalue block. By (Bonhomme and Robin, 2009, Lemma 2), the minimizer is unique up to the signs and order of its columns if, for each pair of distinct columns $\mathbf{h}_s, \mathbf{h}_t$ of $\mathbf{H}$, there exists a pair $(i, j) \in \{(i, j) : |i - j| < k\}$ such that the eigenvalues of $\mathbf{C}^{ij}[\mathbf{X}^{\mathsf{F}}]$ corresponding to $\mathbf{h}_s$ and $\mathbf{h}_t$ are distinct. By the decomposition in Eq. (18), this is equivalent to requiring that

$$\exists (i, j) : |i - j| < k, \quad \text{such that} \quad \tau^4 h_{is} h_{js} \kappa_s \neq \tau^4 h_{it} h_{jt} \kappa_t. \tag{19}$$

We next show that Eq. (19) holds for all $s \neq t$ by considering separately the two cases where s and t either belong to two different sets or where s and t belong to the same set of the partition $\mathcal{I}_1, \ldots, \mathcal{I}_R$.

First, assume that s and t belong to different sets of the partition. We proceed with proof by contraposition and assume that Eq. (19) does not hold. That is, for all (i, j), $|i - j| < k$, the eigenvalue pairs are always equal. In particular, $\tau^4 h_{is}^2 \kappa_s = \tau^4 h_{it}^2 \kappa_t$ for all $i \in \{1, \ldots, p\}$. By summing over the p equations, we have, from the orthogonality of $\mathbf{H}$, that $\tau^4 \kappa_s = \tau^4 \kappa_t$, where $\tau^4 > 0$. This implies that $\kappa_s = \kappa_t$, which is a contradiction since we assumed that s and t belong to different sets of the partition and thus have different eigenvalues. Consequently, Eq. (19) holds for any pair of columns belonging to distinct sets of the index partition.

Assume then that s and t belong to the same set $\mathcal{I}_q$ of the partition. We again proceed with proof by contraposition and assume that Eq. (19) does not hold. Now, $\kappa_s = \kappa_t$ and we have that $\tau^4 h_{is} h_{js} \kappa_s = \tau^4 h_{it} h_{jt} \kappa_s$ for all (i, j), $|i - j| < k$. In particular, this holds for all (i, j) in the subset

$$\{(i, j) : |i - j| < k\} \cap \left\{(i, j) : i \in \mathcal{I}_q \wedge j \in \mathcal{I}_q\right\}. \tag{20}$$

By Assumption 2(v) $k_q \leq k$ and consequently the distance $|i - j|$ is always less than k in the set $\left\{(i, j) : i \in \mathcal{I}_q \wedge j \in \mathcal{I}_q\right\}$. Hereby, the intersection in Eq. (20) is equal to $\left\{(i, j) : i \in \mathcal{I}_q \wedge j \in \mathcal{I}_q\right\}$. We now multiply each equality $\tau^4 h_{is} h_{js} \kappa_s = \tau^4 h_{it} h_{jt} \kappa_s$, with indices (i, j) in the set $\left\{(i, j) : i \in \mathcal{I}_q \wedge j \in \mathcal{I}_q\right\}$, by $h_{is} h_{it}$. By summing twice over $\mathcal{I}_q$, we obtain

$$\tau^4 \kappa_s \left(\sum_{i \in \mathcal{I}_q} h_{is}^2 \right) \left(\sum_{j \in \mathcal{I}_q} h_{js}^2 \right) = \tau^4 \kappa_s \left(\sum_{i \in \mathcal{I}_q} h_{is} h_{it} \right) \left(\sum_{j \in \mathcal{I}_q} h_{js} h_{jt} \right). \tag{21}$$

The constant $\tau^4 > 0$. The constant $\kappa_s \neq 0$, as the contrary would imply that two of the kurtosis values were equal to zero, $\kappa_s = \kappa_t = 0$, which would then contradict Assumption 1. Thus we can divide both sides of Eq. (21) by $\tau^4 \kappa_s$ and we obtain

$$\left(\sum_{i \in \mathcal{I}_q} h_{is}^2 \right) \left(\sum_{j \in \mathcal{I}_q} h_{js}^2 \right) = \left(\sum_{i \in \mathcal{I}_q} h_{is} h_{it} \right) \left(\sum_{j \in \mathcal{I}_q} h_{js} h_{jt} \right). \tag{22}$$

As $\mathbf{H}$ has the block diagonal structure given in Eq. (17), the sums in Eq. (22) are dot products between the columns of the qth orthogonal block of $\mathbf{H}$ and the left-hand side of Eq. (22) is equal to one and the right-hand side of Eq. (22) is equal to zero. This is obviously a contradiction. Consequently, Eq. (19) holds also for any pair of columns which belong to the same set of the partition This concludes the proof of condition *(i)*.

Condition *(ii)*:

To see that condition *(ii)* holds, recall from Virta et al. (2017) that the TFOBI functional $\mathbf{\Gamma}^{\text{F}}$ is orthogonally equivariant and that the TFOBI-transformation is orthogonally invariant. Thus, for all $\mathbf{X} \in \mathcal{R}^{p\times q}$ and all orthogonal $\mathbf{U}_1 \in \mathcal{R}^{p\times p}$, $\mathbf{U}_2 \in \mathcal{R}^{q\times q}$, we have that

$$\mathbf{\Gamma}^k[\mathbf{U}_1\mathbf{X}\mathbf{U}_2'] = \mathbf{V}[(\mathbf{U}_1\mathbf{X}\mathbf{U}_2)^{\text{F}}]\mathbf{\Gamma}^{\text{F}}[\mathbf{U}_1\mathbf{X}\mathbf{U}_2'] \equiv \mathbf{V}[\mathbf{X}^{\text{F}}]\mathbf{\Gamma}^{\text{F}}[\mathbf{X}]\mathbf{U}_1' = \mathbf{\Gamma}^k\,[\mathbf{X}]\,\mathbf{U}_1'.$$

This concludes the proof of condition *(ii)*.

Proof of Theorem 2 Recall that $\mathbf{X}_N = \{\mathbf{X}_1 \ldots \mathbf{X}_n\}$ is an i.i.d. sequence from the tensor IC model and let $\bar{\mathbf{X}}$ be the sample mean of $\mathbf{X}_N$. Then, the TFOBI-transformed observations are

$$\mathbf{X}_i^{\text{F}} = \hat{\mathbf{H}}(\hat{\mathbf{\Sigma}}_1[\mathbf{X}_N])^{-\frac{1}{2}}\,(\mathbf{X}_i - \bar{\mathbf{X}})\,(\hat{\mathbf{\Sigma}}_2[\mathbf{X}_N])^{-\frac{1}{2}}\hat{\mathbf{R}}' = \hat{\mathbf{H}}\mathbf{Y}_i\hat{\mathbf{R}}', \quad i \in \{1,\ldots,n\},$$

where the orthogonal matrices $\hat{\mathbf{H}}, \hat{\mathbf{R}}$ are the left and right TFOBI-rotations and where $(\hat{\mathbf{\Sigma}}_j[\mathbf{X}_N])^{-1/2}, j \in \{1,2\}$, are the symmetric square roots of the left and right sample covariance matrices. Note that the cumulant matrices $\hat{\mathbf{C}}^{ij}$ depend on the observations $\mathbf{X}_i^{\text{F}}$ only through the product

$$\mathbf{X}_i^{\text{F}}(\mathbf{X}_i^{\text{F}})' = \hat{\mathbf{H}}\mathbf{Y}_i\hat{\mathbf{R}}'\hat{\mathbf{R}}\mathbf{Y}_i'\hat{\mathbf{H}}' = \hat{\mathbf{H}}\mathbf{Y}_i\mathbf{Y}_i'\hat{\mathbf{H}}',$$

allowing us to omit the right rotation $\hat{\mathbf{R}}$ in the following.

What makes proving the limiting results challenging, is that the sequence of matrices $\hat{\mathbf{H}}$ has no general limiting properties. If there are any kurtosis values having multiplicity larger than one, this implies that the corresponding eigenvectors are not uniquely defined and, consequently, the TFOBI-solution for them does not converge. However, we can still say two things: First, by the compactness of the set of orthogonal matrices, $\hat{\mathbf{H}} = O_p(1)$. Second, by Lemma 3 and the central limit theorem, the elements of any column of $\hat{\mathbf{H}}$ with indices not belonging to the corresponding diagonal multiplicity block converge to 0 with the rate of root-n. That is, $\sqrt{n}\hat{h}_{kl} = O_p(1)$, for k, l satisfying the aforementioned conditions. These two, in conjunction with Assumption 2, are sufficient for proving the limiting results.

As our first task, we show that the sample and the population objective functions of k-TJADE are asymptotically equivalent to those of TJADE. The left k-TJADE estimator is $\hat{\mathbf{V}}'\hat{\mathbf{H}}(\hat{\mathbf{\Sigma}}_1\,[\mathbf{X}_N])^{-1/2}$, where the orthogonal $\hat{\mathbf{V}} = (\hat{\mathbf{v}}_1,\ldots,\hat{\mathbf{v}}_p)$ is, by Lemma 1, the sequence of the maximizers of the sequence of objective functions,

$$\hat{g}(\mathbf{V},\hat{\mathbf{H}}\mathbf{Y}_N) = \sum_{|i-j|<k}^{p} \left\|\text{diag}\left(\mathbf{V}'\hat{\mathbf{C}}^{ij}[\hat{\mathbf{H}}\mathbf{Y}_N]\mathbf{V}\right)\right\|_{\text{F}}^2 = \sum_{|i-j|<k}^{p}\sum_{l=1}^{p}\left(\mathbf{v}_l'\hat{\mathbf{C}}^{ij}[\hat{\mathbf{H}}\mathbf{Y}_N]\mathbf{v}_l\right)^2.$$

Consistency of the estimator $\hat{\mathbf{V}}'\hat{\mathbf{H}}(\hat{\mathbf{\Sigma}}_1\,[\mathbf{X}_N])^{-1/2}$ is now equal to the claim that there exists a sequence of estimators $\hat{\mathbf{V}}'\hat{\mathbf{H}}(\hat{\mathbf{\Sigma}}_1\,[\mathbf{X}_N])^{-1/2}$ that converges in probability to $\mathbf{I}_p$. It is sufficient to show that $\hat{\mathbf{V}}'\hat{\mathbf{H}} \to_{\mathbb{P}} \mathbf{I}_p$, since the weak law of large numbers and the continuous mapping theorem directly imply that $(\hat{\mathbf{\Sigma}}_1\,[\mathbf{X}_N])^{-1/2} \to_{\mathbb{P}} \mathbf{I}_p$.

Since $\hat{\mathbf{V}}$ maximizes the objective function $\hat{g}$ and since $\hat{\mathbf{H}}$ is orthogonal, we have that

$$\hat{g}(\hat{\mathbf{V}}, \hat{\mathbf{H}}\mathbf{Y}_N) \geq \hat{g}(\mathbf{V}, \hat{\mathbf{H}}\mathbf{Y}_N), \quad \forall\, \mathbf{V} \text{ orthogonal}, \quad \text{and}$$
$$\hat{g}(\hat{\mathbf{H}}\hat{\mathbf{H}}'\hat{\mathbf{V}}, \hat{\mathbf{H}}\mathbf{Y}_N) \geq \hat{g}(\hat{\mathbf{H}}\hat{\mathbf{H}}'\mathbf{V}, \hat{\mathbf{H}}\mathbf{Y}_N), \quad \forall\, \mathbf{V} \text{ orthogonal}.$$

The range of $\mathbf{V} \mapsto \hat{\mathbf{H}}'\mathbf{V}$ is the set of all $p \times p$ orthogonal matrices and consequently $\hat{\mathbf{H}}'\hat{\mathbf{V}} = \hat{\mathbf{W}}$ is the sequence of maximizers for the argument-modified sequence of objective functions, $\hat{g}(\hat{\mathbf{H}}\mathbf{W}, \hat{\mathbf{H}}\mathbf{Y}_N)$. The original sequence of maximizers can be written as $\hat{\mathbf{V}} = \hat{\mathbf{H}}\hat{\mathbf{W}}$ and the claim takes the form $\hat{\mathbf{W}} \to_{\mathbb{P}} \mathbf{I}_p$. Applying Lemma 2, this new sequence of objective functions $\hat{g}(\hat{\mathbf{H}}\mathbf{W}, \hat{\mathbf{H}}\mathbf{Y}_N)$ can be reformulated as

$$\begin{aligned}\hat{g}(\hat{\mathbf{H}}\mathbf{W}, \hat{\mathbf{H}}\mathbf{Y}_N) &= \sum_{|i-j|<k}^{p} \sum_{l=1}^{p} \left(\mathbf{w}_l'\hat{\mathbf{H}}'\hat{\mathbf{C}}^{ij}[\hat{\mathbf{H}}\mathbf{Y}_N]\hat{\mathbf{H}}\mathbf{w}_l\right)^2 \\ &= \sum_{|i-j|<k}^{p} \sum_{l=1}^{p} \sum_{a,b} \sum_{a',b'} \hat{h}_{ia}\hat{h}_{jb}\hat{h}_{ia'}\hat{h}_{jb'}\mathbf{w}_l'\hat{\mathbf{C}}^{ab}[\mathbf{Y}_N]\mathbf{w}_l\mathbf{w}_l'\hat{\mathbf{C}}^{a'b'}[\mathbf{Y}_N]\mathbf{w}_l.\end{aligned}$$

Next, we add and subtract,

$$\hat{m} = \sum_{|i-j|\geq k}^{p} \sum_{l=1}^{p} \sum_{a,b} \sum_{a',b'} \hat{h}_{ia}\hat{h}_{jb}\hat{h}_{ia'}\hat{h}_{jb'}\mathbf{w}_l'\hat{\mathbf{C}}^{ab}[\mathbf{Y}_N]\mathbf{w}_l\mathbf{w}_l'\hat{\mathbf{C}}^{a'b'}[\mathbf{Y}_N]\mathbf{w}_l \tag{23}$$

in order to make the first sum run over the whole range of i and j. This gives us

$$\begin{aligned}\hat{g}(\hat{\mathbf{H}}\mathbf{W}, \hat{\mathbf{H}}\mathbf{Y}_N) &= \sum_{i=1}^{p} \sum_{j=1}^{p} \sum_{l=1}^{p} \sum_{a,b} \sum_{a',b'} \hat{h}_{ia}\hat{h}_{jb}\hat{h}_{ia'}\hat{h}_{jb'}\mathbf{w}_l'\hat{\mathbf{C}}^{ab}[\mathbf{Y}_N]\mathbf{w}_l\mathbf{w}_l'\hat{\mathbf{C}}^{a'b'}[\mathbf{Y}_N]\mathbf{w}_l - \hat{m} \\ &= \sum_{l=1}^{p} \sum_{a,b} \sum_{a',b'} \sum_{i=1}^{p} \left(\hat{h}_{ia}\hat{h}_{ia'}\right) \sum_{j=1}^{p} \left(\hat{h}_{jb}\hat{h}_{jb'}\right) \mathbf{w}_l'\hat{\mathbf{C}}^{ab}[\mathbf{Y}_N]\mathbf{w}_l\mathbf{w}_l'\hat{\mathbf{C}}^{a'b'}[\mathbf{Y}_N]\mathbf{w}_l - \hat{m} \\ &= \sum_{l=1}^{p} \sum_{a,b} \sum_{a',b'} \delta_{aa'}\delta_{bb'}\mathbf{w}_l'\hat{\mathbf{C}}^{ab}[\mathbf{Y}_N]\mathbf{w}_l\mathbf{w}_l'\hat{\mathbf{C}}^{a'b'}[\mathbf{Y}_N]\mathbf{w}_l - \hat{m} \\ &= \sum_{l=1}^{p} \sum_{a,b} \mathbf{w}_l'\hat{\mathbf{C}}^{ab}[\mathbf{Y}_N]\mathbf{w}_l\mathbf{w}_l'\hat{\mathbf{C}}^{ab}[\mathbf{Y}_N]\mathbf{w}_l - \hat{m} \\ &= \sum_{a,b} \sum_{l=1}^{p} \left(\mathbf{w}_l'\hat{\mathbf{C}}^{ab}[\mathbf{Y}_N]\mathbf{w}_l\right)^2 - \hat{m},\end{aligned}$$

where the third equality follows from the orthogonality of $\hat{\mathbf{H}}$.

Next, we establish two properties of $\hat{m}$: (1°) If the argument $\mathbf{W}$ is replaced with any sequence of orthogonal matrices bounded in probability (the boundedness is an instant consequence of the orthogonality), $n \cdot \hat{m}$ is bounded in probability. (2°) $\hat{m}$ converges uniformly in probability to zero.

We first prove the property (1°) (which will be used in the proof of Theorem 3 later). Let

$$\tilde{m} = \sum_{|i-j|\geq k}^{p} \sum_{l=1}^{p} \sum_{a,b} \sum_{a',b'} \hat{h}_{ia}\hat{h}_{jb}\hat{h}_{ia'}\hat{h}_{jb'}\tilde{\mathbf{w}}_l'\hat{\mathbf{C}}^{ab}[\mathbf{Y}_N]\tilde{\mathbf{w}}_l\tilde{\mathbf{w}}_l'\hat{\mathbf{C}}^{a'b'}[\mathbf{Y}_N]\tilde{\mathbf{w}}_l,$$

where $\tilde{\mathbf{W}} = (\tilde{\mathbf{w}}_1, \dots, \tilde{\mathbf{w}}_p)$ is some sequence of orthogonal matrices bounded in probability and all other terms are as in the definition of $\hat{m}$ in Eq. (23). We divide the terms of $\tilde{m}$ into different cases based on the indices.

1. $a \neq b$, $a' \neq b'$: By the supplementary material of Virta et al. (2018) and by the central limit theorem that $\hat{\mathbf{C}}^{ab}[\mathbf{Y}_N] = O_p(1/\sqrt{n})$ and that $\hat{\mathbf{C}}^{a'b'}[\mathbf{Y}_N] = O_p(1/\sqrt{n})$. As the elements $\hat{h}_{ia}, \hat{h}_{jb}, \hat{h}_{ia'}, \hat{h}_{jb'}, \tilde{\mathbf{w}}_l$ are bounded in probability for all indices i, j, a, b, a', b', l, any summand in $\tilde{m}$ with the indices $a \neq b$ and $a' \neq b'$ is $O_p(1/n)$.
2. $a \neq b$, $a' = b'$: By the same arguments as in case 1, we have that $\hat{\mathbf{C}}^{ab}[\mathbf{Y}_N] = O_p(1/\sqrt{n})$. Furthermore, the supplementary material of Virta et al. (2018) provides us the result $\hat{\mathbf{C}}^{a'b'}[\mathbf{Y}_N] \to_{\mathbb{P}} \mathbf{C}^{a'b'}$ for some constant matrix $\mathbf{C}^{a'b'}$. Both $\hat{h}_{ia'}$ and $\hat{h}_{ja'}$ (with $|i - j| \geq k$) cannot belong to a diagonal block of $\hat{\mathbf{H}}$ as that would compromise Assumption 2(v). Thus, by Lemma 3, at least one of $\hat{h}_{ia'}$ and $\hat{h}_{ja'}$ must be $O_p(1/\sqrt{n})$. Consequently, any summand in $\tilde{m}$ with the indices $a \neq b$ and $a' = b'$ is $O_p(1/n)$.
3. $a = b$, $a' \neq b'$: Since $\tilde{m}$ is symmetric with respect to (a, b) and (a', b'), it follows from the same arguments as in case 2 that any summand in $\tilde{m}$ with the indices $a = b$ and $a' \neq b'$ is $O_p(1/n)$.
4. $a = b$, $a' = b'$: By the same arguments as above, $\hat{\mathbf{C}}^{ab}[\mathbf{Y}_N]$ and $\hat{\mathbf{C}}^{a'b'}[\mathbf{Y}_N]$ both converge in probability to some constant matrices and the pairs $(\hat{h}_{ia'}, \hat{h}_{ja'})$ and $(\hat{h}_{ia}, \hat{h}_{ja})$ each provide one term that is $O_p(1/\sqrt{n})$. Thus any summand in $\tilde{m}$ with the indices $a = b$ and $a' = b'$ is $O_p(1/n)$.

Since the sum defining $\tilde{m}$ is a finite sum of $O_p(1/n)$-terms, it holds that $\tilde{m} = O_p(1/n)$. Note that by choosing $\tilde{\mathbf{W}} = \mathbf{W}$, we have $\tilde{m} = \hat{m}$ and the objective function satisfies

$$\hat{g}(\hat{\mathbf{H}}\mathbf{W}, \hat{\mathbf{H}}\mathbf{Y}_N) = \sum_{a,b}^{p} \sum_{l=1}^{p} \left(\mathbf{w}_l' \hat{\mathbf{C}}^{ab}[\mathbf{Y}_N] \mathbf{w}_l \right)^2 + O_p(1/n) = \hat{f}(\mathbf{W}, \mathbf{Y}_N) + O_p(1/n),$$

where $\hat{f}(\mathbf{W}, \mathbf{Y}) = \sum_{a,b}^{p} \sum_{l=1}^{p} (\mathbf{w}_l' \hat{\mathbf{C}}^{ab}[\mathbf{Y}] \mathbf{w}_l)^2$.
We next prove property (2°). By the triangle inequality and the monotonicity of supremum,

$$\sup_{\mathbf{W} \in \mathcal{U}} \{|\hat{m}|\} \leq \sum_{|i-j| \geq k}^{p} \sum_{l=1}^{p} \sum_{a,b} \sum_{a',b'} \sup_{\mathbf{W} \in \mathcal{U}} \left\{ \left| \hat{h}_{ia} \hat{h}_{jb} \hat{h}_{ia'} \hat{h}_{jb'} \mathbf{w}_l' \hat{\mathbf{C}}^{ab}[\mathbf{Y}_N] \mathbf{w}_l \mathbf{w}_l' \hat{\mathbf{C}}^{a'b'}[\mathbf{Y}_N] \mathbf{w}_l \right| \right\},$$

where $\mathcal{U} = \{\mathbf{V} \in \mathcal{R}^{p \times p} : \mathbf{V}\mathbf{V}' = \mathbf{I}_p\}$. It is thus sufficient to show that

$$\sup_{\mathbf{W} \in \mathcal{U}} \left\{ \left| \hat{h}_{ia} \hat{h}_{jb} \hat{h}_{ia'} \hat{h}_{jb'} \mathbf{w}_l' \hat{\mathbf{C}}^{ab}[\mathbf{Y}_N] \mathbf{w}_l \mathbf{w}_l' \hat{\mathbf{C}}^{a'b'}[\mathbf{Y}_N] \mathbf{w}_l \right| \right\} = o_p(1)$$

individually for all terms in the sum $\hat{m}$. By the Cauchy-Schwarz inequality, the operator norm inequality, the equivalence of all finite-dimensional norms and the monotonicity of the supremum, we obtain the following upper bound (where the dependency of the expression on the variable $\mathbf{W}$ is removed by the unit lengths of its columns),

$$\begin{aligned}
&\sup_{\mathbf{W} \in \mathcal{U}} \left\{ \left| \hat{h}_{ia} \hat{h}_{jb} \hat{h}_{ia'} \hat{h}_{jb'} \mathbf{w}_l' \hat{\mathbf{C}}^{ab}[\mathbf{Y}_N] \mathbf{w}_l \mathbf{w}_l' \hat{\mathbf{C}}^{a'b'}[\mathbf{Y}_N] \mathbf{w}_l \right| \right\} \\
&\leq \sup_{\mathbf{W} \in \mathcal{U}} \left\{ |\hat{h}_{ia} \hat{h}_{jb} \hat{h}_{ia'} \hat{h}_{jb'}| \|\mathbf{w}_l\|_F \|\hat{\mathbf{C}}^{ab}[\mathbf{Y}_N] \mathbf{w}_l\|_F \|\mathbf{w}_l\|_F \|\hat{\mathbf{C}}^{a'b'}[\mathbf{Y}_N] \mathbf{w}_l\|_F \right\} \\
&\leq \sup_{\mathbf{W} \in \mathcal{U}} \left\{ |\hat{h}_{ia} \hat{h}_{jb} \hat{h}_{ia'} \hat{h}_{jb'}| \|\hat{\mathbf{C}}^{ab}[\mathbf{Y}_N]\|_2 \|\mathbf{w}_l\|_F \|\hat{\mathbf{C}}^{a'b'}[\mathbf{Y}_N]\|_2 \|\mathbf{w}_l\|_F \right\} \\
&\leq c_0 |\hat{h}_{ia} \hat{h}_{jb} \hat{h}_{ia'} \hat{h}_{jb'}| \|\hat{\mathbf{C}}^{ab}[\mathbf{Y}_N]\|_F \|\hat{\mathbf{C}}^{a'b'}[\mathbf{Y}_N]\|_F,
\end{aligned} \tag{24}$$

where c_0 is a constant resulting from switching from the operator norm to the Frobenius norm. Using the same arguments we used to prove the property ($1°$) above, Eq. (24) can be seen to always converge in probability to zero, regardless of the values of the indices. Thus $\hat{g}(\hat{\mathbf{H}}\mathbf{W}, \hat{\mathbf{H}}\mathbf{Y}_N) = \hat{f}(\mathbf{W}, \mathbf{Y}_N) - \hat{m}$ for which $\sup_{\mathbf{W}}\{|\hat{m}|\} = o_p(1)$.

Now, Lemma 2 along with exactly analogous calculations as above (with exact zeros taking the roles of $o_p(1)$-terms) can be used to show that under Assumption 2(v), the population version of the argument-modified k-TJADE objective function,

$$g(\mathbf{HW}, \mathbf{HY}) = \sum_{|i-j|<k}^{p} \sum_{l=1}^{p} \left(\mathbf{w}_l' \mathbf{H}' \mathbf{C}^{ij}[\mathbf{HY}] \mathbf{H} \mathbf{w}_l \right)^2,$$

where $\mathbf{H}$ is the population TFOBI-rotation, equals the augmented objective function not depending on $\mathbf{H}$, that is,

$$f(\mathbf{W}, \mathbf{Y}) := \sum_{i,j}^{p} \sum_{l=1}^{p} \left(\mathbf{w}_l' \mathbf{C}^{ij}[\mathbf{Y}] \mathbf{w}_l \right)^2 = g(\mathbf{HW}, \mathbf{HY}).$$

Consequently,

$$\hat{g}(\hat{\mathbf{H}}\mathbf{W}, \hat{\mathbf{H}}\mathbf{Y}_N) - g(\mathbf{HW}, \mathbf{HY}) = \hat{f}(\mathbf{W}, \mathbf{Y}_N) - f(\mathbf{W}, \mathbf{Y}) - \hat{m}, \tag{25}$$

where $\hat{f}(\mathbf{W}, \mathbf{Y}_N)$ and $f(\mathbf{W}, \mathbf{Y})$ are the sample and population objective functions of TJADE (Virta et al., 2018).

Having established this, we next show that the difference in the population and the sample k-TJADE objective functions converges uniformly in probability to zero, allowing us to use the M-estimator convergence argument, see e.g. (Van der Vaart, 1998, Theorem 5.7), to prove the consistency of the estimator. By Eq. (25), we have

$$\begin{aligned} &\sup_{\mathbf{W}\in\mathcal{U}} \left\{ \left| \hat{g}(\hat{\mathbf{H}}\mathbf{W}, \hat{\mathbf{H}}\mathbf{Y}_N) - g(\mathbf{HW}, \mathbf{HY}) \right| \right\} \\ = &\sup_{\mathbf{W}\in\mathcal{U}} \left\{ \left| \hat{f}(\mathbf{W}, \mathbf{Y}_N) - f(\mathbf{W}, \mathbf{Y}) - \hat{m} \right| \right\} \\ \leq &\sup_{\mathbf{W}\in\mathcal{U}} \left\{ \left| \hat{f}(\mathbf{W}, \mathbf{Y}_N) - f(\mathbf{W}, \mathbf{Y}) \right| \right\} + o_p(1), \end{aligned} \tag{26}$$

and the uniform convergence of the k-TJADE objective functions is a direct consequence of the uniform convergence of the TJADE objective functions. The latter result is given implicitly in Virta et al. (2018), but for completeness, we present the proof here.

Let,

$$\mathbf{A}^{ij} = \mathrm{diag}\left(\mathbf{W}' \mathbf{C}^{ij}[\mathbf{Y}] \mathbf{W} \right) \quad \text{and} \quad \hat{\mathbf{A}}^{ij} = \mathrm{diag}\left(\mathbf{W}' \hat{\mathbf{C}}^{ij}[\mathbf{Y}_N] \mathbf{W} \right).$$

Since the Frobenius norm of the diagonal elements of a matrix is always bounded by the Frobenius norm of the entire matrix and since $\mathbf{W}$ is orthogonal, the following inequalities hold,

$$\begin{aligned} &\|\mathbf{A}^{ij}\|_F \leq \|\mathbf{C}^{ij}[\mathbf{Y}]\|_F, \quad \|\hat{\mathbf{A}}^{ij}\|_F \leq \|\hat{\mathbf{C}}^{ij}[\mathbf{Y}_N]\|_F \quad \text{and} \\ &\left\| \mathbf{A}^{ij} - \hat{\mathbf{A}}^{ij} \right\|_F = \left\| \mathrm{diag}\left(\mathbf{W}'(\mathbf{C}^{ij}[\mathbf{Y}] - \hat{\mathbf{C}}^{ij}[\mathbf{Y}_N]) \mathbf{W} \right) \right\|_F \leq \left\| \mathbf{C}^{ij}[\mathbf{Y}] - \hat{\mathbf{C}}^{ij}[\mathbf{Y}_N] \right\|_F. \end{aligned}$$

By the reverse triangle inequality, we have that for any $\mathbf{A}, \mathbf{B} \in \mathcal{R}^{p\times p}$,

$$\left| \|\mathbf{A}\|_F^2 - \|\mathbf{B}\|_F^2 \right| = |(\|\mathbf{A}\|_F - \|\mathbf{B}\|_F)(\|\mathbf{A}\|_F + \|\mathbf{B}\|_F)| \leq \|\mathbf{A} - \mathbf{B}\|_F \left(\|\mathbf{A}\|_F + \|\mathbf{B}\|_F\right).$$

Now, by the monotonicity of the supremum,

$$\begin{aligned}\sup_{\mathbf{W}\in\mathcal{U}} \left\{ |f(\mathbf{W},\mathbf{Y}) - \hat{f}(\mathbf{W},\mathbf{Y}_N)| \right\} &\leq \sup_{\mathbf{W}\in\mathcal{U}} \left\{ \sum_{i,j}^{p} \left| \|\mathbf{A}^{ij}\|_F^2 - \|\hat{\mathbf{A}}^{ij}\|_F^2 \right| \right\} \\ &\leq \sup_{\mathbf{W}\in\mathcal{U}} \left\{ \sum_{i,j}^{p} \left\| \mathbf{A}^{ij} - \hat{\mathbf{A}}^{ij} \right\|_F \left(\|\mathbf{A}^{ij}\|_F + \|\hat{\mathbf{A}}^{ij}\|_F \right) \right\} \\ &\leq \sum_{i,j}^{p} \left\| \mathbf{C}^{ij}[\mathbf{Y}] - \hat{\mathbf{C}}^{ij}[\mathbf{Y}_N] \right\|_F \left(\|\mathbf{C}^{ij}[\mathbf{Y}]\|_F + \|\hat{\mathbf{C}}^{ij}[\mathbf{Y}_N]\|_F \right),\end{aligned} \tag{27}$$

which converges in probability to zero, since $\hat{\mathbf{C}}^{ij}[\mathbf{Y}_N] \to_{\mathbb{P}} \mathbf{C}^{ij}[\mathbf{Y}]$. Thus, the sequence of the sample TJADE objective functions $\hat{f}(\mathbf{W}, \mathbf{Y}_N)$ converges uniformly in probability, with respect to the set of orthogonal matrices, to the theoretical TJADE objective function $f(\mathbf{W}, \mathbf{Y})$. It now follows from Eqs. (26) and (27), that under Assumption 1 and Assumption 2(v), the same holds for the sequence of the sample k-TJADE objective functions.

We close the proof with the M-estimator convergence argument. In the following we use the notation, $h(\mathbf{W}, \mathbf{Y}) := g(\mathbf{HW}, \mathbf{HY})$ and $\hat{h}(\mathbf{W}, \mathbf{Y}_N) := \hat{g}(\hat{\mathbf{H}}\mathbf{W}, \hat{\mathbf{H}}\mathbf{Y}_N)$. As all our maximizers are unique only up to the signs and the order of their columns, to obtain a sequence such that $\hat{\mathbf{W}} \to_{\mathbb{P}} \mathbf{I}_p$, we restrict ourselves to a subset $\mathcal{U}_0$ of $\mathcal{U}$ where the signs and order are fixed and $\mathbf{W} = \mathbf{I}_p$ is the unique maximizer of $h(\mathbf{W}, \mathbf{Y})$. A corresponding set $\mathcal{U}_0$ can be constructed as follows,

$$\mathcal{U}_0 = \left\{ \mathbf{W} \in \mathcal{R}^{p\times p} : (\mathbf{W} \in \mathcal{U}) \wedge (\mathbf{w}_i' \mathbf{1}_p \geq 0, \forall i) \wedge \left(\mathbf{w}_1' \mathbf{D}^{11} \mathbf{w}_1 \geq \ldots \geq \mathbf{w}_p' \mathbf{D}^{pp} \mathbf{w}_p \right) \right\},$$

where $\mathbf{D}^{ii} = (p-1)\mathbf{E}^{ii}$. Moreover, as the conditions defining $\mathcal{U}_0$ are continuous in $\mathbf{W}$, there exists $\varepsilon > 0$ such that an ε-neighborhood around $\mathbf{I}_p$ fits completely within $\mathcal{U}_0$. The following set defines the complement of such ball in $\mathcal{U}_0$,

$$\mathcal{U}_\varepsilon = \left\{ \mathbf{W} \in \mathcal{U}_0 : \left\| \mathbf{W} - \mathbf{I}_p \right\|_F \geq \varepsilon > 0 \right\}.$$

Since there is a finite number of different combinations of the order and the signs of the columns of $\hat{\mathbf{W}}$, we can, for every n, consider the equivalent maximizer $\hat{\mathbf{W}} \equiv \mathbf{PJ}\hat{\mathbf{W}}$ that belongs to the set $\mathcal{U}_0$, i.e. $\hat{\mathbf{W}} = \text{argmax}_{\mathbf{W}\in\mathcal{U}_0} \{\hat{h}(\mathbf{W}, \mathbf{Y}_N)\}$. Furthermore, by the definition of $\mathcal{U}_0$, the unique maximizer $\text{argmax}_{\mathbf{W}\in\mathcal{U}_0} \{h(\mathbf{W}, \mathbf{Y})\} = \mathbf{I}_p$.

Now,

$$\hat{h}(\hat{\mathbf{W}}, \mathbf{Y}_N) \geq \hat{h}(\mathbf{I}_p, \mathbf{Y}_N) - h(\mathbf{I}_p, \mathbf{Y}) + h(\mathbf{I}_p, \mathbf{Y}),$$

and by subtracting $h(\hat{\mathbf{W}}, \mathbf{Y})$ from both sides and applying Eq. (27), we obtain,

$$\begin{aligned} 0 \leq h(\mathbf{I}_p, \mathbf{Y}) - h(\hat{\mathbf{W}}, \mathbf{Y}) &\leq \hat{h}(\hat{\mathbf{W}}, \mathbf{Y}_N) - h(\hat{\mathbf{W}}, \mathbf{Y}) + h(\mathbf{I}_p, \mathbf{Y}) - \hat{h}(\mathbf{I}_p, \mathbf{Y}_N) \\ &\leq 2 \sup_{\mathbf{W}\in\mathcal{U}_0} \left\{ \left| \hat{h}(\mathbf{W}, \mathbf{Y}_N) - h(\mathbf{W}, \mathbf{Y}) \right| \right\} = o_p(1). \end{aligned} \tag{28}$$

Furthermore, it follows from the uniqueness of the maximizer $\mathbf{I}_p$ of h in $\mathcal{U}_0$, that, for every $\varepsilon > 0$, there exists $\delta > 0$,

such that

$$\sup_{\mathbf{W} \in \mathcal{U}_\varepsilon} \{h(\mathbf{W}, \mathbf{Y})\} < h(\mathbf{I}_p, \mathbf{Y}) - \delta.$$

Fix now $\varepsilon > 0$ small enough along with the corresponding $\delta > 0$. Then,

$$\{\hat{\mathbf{W}} \in \mathcal{U}_\varepsilon\} \subset \{h(\mathbf{I}_p, \mathbf{Y}) - h(\hat{\mathbf{W}}, \mathbf{Y}) > \delta\},$$

and

$$\mathbb{P}\left[\|\hat{\mathbf{W}} - \mathbf{I}_p\|_{\mathrm{F}} \geq \varepsilon\right] = \mathbb{P}\left[\hat{\mathbf{W}} \in \mathcal{U}_\varepsilon\right] \leq \mathbb{P}\left[\left\{h(\mathbf{I}_p, \mathbf{Y}) - h(\hat{\mathbf{W}}, \mathbf{Y}) > \delta\right\}\right] \underset{n\to\infty}{\longrightarrow} 0,$$

where the convergence follows from Eq. (28). Thus, $\hat{\mathbf{W}} \to_{\mathbb{P}} \mathbf{I}_p$. This concludes the proof.

Proof of Theorem 3 As stated in the proof of Theorem 2, the estimated left k-TJADE transformation is $\hat{\mathbf{\Gamma}}^k = \hat{\mathbf{V}}'\hat{\mathbf{H}}\hat{\mathbf{\Sigma}}_1^{-1/2} = \hat{\mathbf{W}}'\hat{\mathbf{\Sigma}}_1^{-1/2}$, where $\hat{\mathbf{W}} = (\hat{\mathbf{w}}_1, \ldots, \hat{\mathbf{w}}_p)$ is the sequence of the maximizers of $\hat{g}(\hat{\mathbf{H}}\mathbf{W}, \hat{\mathbf{H}}\mathbf{Y}_N)$. It follows from linearization and the consistency of $\hat{\mathbf{W}}$ that,

$$\begin{aligned}\sqrt{n}(\hat{\mathbf{\Gamma}}^k - \mathbf{I}_p) &= \hat{\mathbf{W}}'\sqrt{n}(\hat{\mathbf{\Sigma}}_1^{-1/2} - \mathbf{I}_p) + \sqrt{n}(\hat{\mathbf{W}}' - \mathbf{I}_p)\\ &= \sqrt{n}(\hat{\mathbf{\Sigma}}_1^{-1/2} - \mathbf{I}_p) + \sqrt{n}(\hat{\mathbf{W}}' - \mathbf{I}_p) + o_p(1).\end{aligned}$$

As the covariance standardization part is the same in both k-TJADE and TJADE, our goal in the following is to show that the expression $\sqrt{n}(\hat{\mathbf{W}}' - \mathbf{I}_p)$ is asymptotically equivalent to $\sqrt{n}(\hat{\mathbf{W}}_{\mathrm{J}}' - \mathbf{I}_p)$, where $\hat{\mathbf{W}}_{\mathrm{J}}$ is the regular TJADE-rotation. The estimating equations for $\hat{\mathbf{W}}$ can be found by applying the technique of Lagrangian multipliers, under the constraint $\mathbf{W}'\mathbf{W} = \mathbf{I}_p$, to the objective function $\hat{g}(\hat{\mathbf{H}}\mathbf{W}, \hat{\mathbf{H}}\mathbf{Y}_N)$. Similarly as in Miettinen et al. (2016), the estimating equations are

$$\hat{\mathbf{w}}_l'\hat{\mathbf{T}}(\hat{\mathbf{w}}_m) = \hat{\mathbf{w}}_m'\hat{\mathbf{T}}(\hat{\mathbf{w}}_l),$$

for any two distinct columns of $\hat{\mathbf{W}}$, $m \neq l$, where

$$\begin{aligned}\hat{\mathbf{T}}(\hat{\mathbf{w}}_m) &= \sum_{|i-j|<k} \hat{\mathbf{H}}'\hat{\mathbf{C}}^{ij}[\hat{\mathbf{H}}\mathbf{Y}_N]\hat{\mathbf{H}}\hat{\mathbf{w}}_m\hat{\mathbf{w}}_m'\hat{\mathbf{H}}'\hat{\mathbf{C}}^{ij}[\hat{\mathbf{H}}\mathbf{Y}_N]\hat{\mathbf{H}}\hat{\mathbf{w}}_m\\ &= \sum_{|i-j|<k}\sum_{a,b}\sum_{a',b'} \hat{h}_{ia}\hat{h}_{jb}\hat{h}_{ia'}\hat{h}_{jb'}\hat{\mathbf{C}}^{ab}[\mathbf{Y}_N]\hat{\mathbf{w}}_m\hat{\mathbf{w}}_m'\hat{\mathbf{C}}^{a'b'}[\mathbf{Y}_N]\hat{\mathbf{w}}_m\\ &= \sum_{a,b}\hat{\mathbf{C}}^{ab}[\mathbf{Y}_N]\hat{\mathbf{w}}_m\hat{\mathbf{w}}_m'\hat{\mathbf{C}}^{ab}[\mathbf{Y}_N]\hat{\mathbf{w}}_m - \hat{\mathbf{m}}.\end{aligned} \tag{29}$$

In the above formula, the vector $\hat{\mathbf{m}}$ is defined as,

$$\hat{\mathbf{m}} = \sum_{|i-j|\geq k}\sum_{a,b}\sum_{a',b'} \hat{h}_{ia}\hat{h}_{jb}\hat{h}_{ia'}\hat{h}_{jb'}\hat{\mathbf{C}}^{ab}[\mathbf{Y}_N]\hat{\mathbf{w}}_m\hat{\mathbf{w}}_m'\hat{\mathbf{C}}^{a'b'}[\mathbf{Y}_N]\hat{\mathbf{w}}_m, \tag{30}$$

the second equality is based on Lemma 2 and the third equality is based on the sum-completion technique that we applied also in the proof of Theorem 2. Under Assumption 2(v), the vector $\hat{\mathbf{m}}$ satisfies $\hat{\mathbf{m}} = O_p(1/n)$. We omit the

proof, since it is almost identical to the proof of property (1°) in the proof of Theorem 2.

We denote $\mathbf{C}^{ab} := \mathbf{C}^{ab}[\mathbf{Y}]$ and $\hat{\mathbf{C}}^{ab} := \hat{\mathbf{C}}^{ab}[\mathbf{Y}_N]$. Then, by Eq. (29) and Eq. (30), any solution to the ($\sqrt{n}$-multiplied) k-TJADE estimating equations,

$$\sqrt{n}\hat{\mathbf{w}}_l'\hat{\mathbf{T}}(\hat{\mathbf{w}}_m) = \sqrt{n}\hat{\mathbf{w}}_m'\hat{\mathbf{T}}(\hat{\mathbf{w}}_l), \quad m \neq l,$$

is also a solution to the estimating equations,

$$\sqrt{n}\sum_{a,b}\hat{\mathbf{w}}_l'\hat{\mathbf{C}}^{ab}\hat{\mathbf{w}}_m\hat{\mathbf{w}}_m'\hat{\mathbf{C}}^{ab}\hat{\mathbf{w}}_m = \sqrt{n}\sum_{a,b}\hat{\mathbf{w}}_m'\hat{\mathbf{C}}^{ab}\hat{\mathbf{w}}_l\hat{\mathbf{w}}_l'\hat{\mathbf{C}}^{ab}\hat{\mathbf{w}}_l + O_p(1/\sqrt{n}), \tag{31}$$

for $m \neq l$, and vice versa. (Note that we may also include the cases where $m = l$ as those simply correspond to the trivial case $0 = o_p(1)$.) These are the estimating equations of the regular TJADE-rotation $\hat{\mathbf{W}}_\mathrm{J}$, and yield a limiting distribution under Assumption 1. It now follows that the limiting expression for $\hat{\mathbf{W}}$ is asymptotically equivalent to the limiting expression of the TJADE-rotation $\hat{\mathbf{W}}_\mathrm{J}$ and, consequently, under Assumption 1 and Assumption 2(v) we have that

$$\sqrt{n}(\hat{\mathbf{\Gamma}}^k - \mathbf{I}_p) = \sqrt{n}(\hat{\mathbf{\Gamma}}^\mathrm{J} - \mathbf{I}_p) + o_p(1),$$

technically concluding the proof. However, as the derivation of the convergence rate, root-n, is left implicit in Virta et al. (2018), we present it here for completeness.

Letting $\hat{\mathbf{D}}^{ab} = \hat{\mathbf{W}}'\hat{\mathbf{C}}^{ab}\hat{\mathbf{W}} \to_{\mathbb{P}} \delta_{ab}\kappa_a\mathbf{E}^{aa}$, the estimating equations in Eq. (31) along with the orthogonality constraint can be written in matrix form as,

$$\sqrt{n}\sum_{a,b}\hat{\mathbf{W}}'\hat{\mathbf{C}}^{ab}\hat{\mathbf{W}}\mathrm{diag}(\hat{\mathbf{D}}^{ab}) = \sqrt{n}\sum_{a,b}\mathrm{diag}(\hat{\mathbf{D}}^{ab})\hat{\mathbf{W}}'\hat{\mathbf{C}}^{ab}\hat{\mathbf{W}} + o_p(1), \tag{32}$$

$$\sqrt{n}(\hat{\mathbf{W}}'\hat{\mathbf{W}} - \mathbf{I}_p) = \mathbf{0}. \tag{33}$$

In order to prove that the convergence rate is root-n, we bring the above estimating equations to the forms,

$$\begin{aligned} \hat{\mathbf{A}}_1\sqrt{n}\mathrm{vec}(\hat{\mathbf{W}} - \mathbf{I}_p) &= O_p(1), \\ \hat{\mathbf{A}}_2\sqrt{n}\mathrm{vec}(\hat{\mathbf{W}} - \mathbf{I}_p) &= O_p(1), \end{aligned} \tag{34}$$

where $\hat{\mathbf{A}}_1, \hat{\mathbf{A}}_2$ converge in probability to some constant matrices $\mathbf{A}_1, \mathbf{A}_2$ and the right-hand sides have limiting distributions depending only on the matrices $\hat{\mathbf{C}}^{ab}$, $a, b \in \{1, \ldots, p\}$. By the central limit theorem, the limiting distributions of $\sqrt{n}(\hat{\mathbf{C}}^{ab} - \mathbf{C}^{ab})$ are multivariate normal. We also show that while neither $\mathbf{A}_1$ nor $\mathbf{A}_2$ is invertible, their sum is, and we may take the sum of the two equations in Eq. (34) and multiply both sides of the resulting equation by $(\hat{\mathbf{A}}_1 + \hat{\mathbf{A}}_2)^{-1}$ from the left. Applying the continuous mapping theorem and Slutsky's theorem then gives us the desired limiting result.

We begin with the orthogonality constraint in Eq. (33) and expand as

$$\mathbf{0} = \sqrt{n}(\hat{\mathbf{W}}'\hat{\mathbf{W}} - \mathbf{I}_p) = \sqrt{n}(\hat{\mathbf{W}}' - \mathbf{I}_p)\hat{\mathbf{W}} + \sqrt{n}(\hat{\mathbf{W}} - \mathbf{I}_p).$$

Applying the vectorization operator along with the identity $\text{vec}(\mathbf{AXB}') = (\mathbf{B}\otimes\mathbf{A})\text{vec}(\mathbf{X})$ gives the first desired equation,

$$\left[(\hat{\mathbf{W}}' \otimes \mathbf{I}_p)\mathbf{K} + \mathbf{I}_{p^2}\right]\sqrt{n}\text{vec}(\hat{\mathbf{W}} - \mathbf{I}_p) = \mathbf{0},$$

where $\mathbf{K} = \sum_{k=1}^{p}\sum_{\ell=1}^{p}\mathbf{E}^{k\ell}\otimes\mathbf{E}^{\ell k}$ is the commutation matrix. Note that $\mathbf{K}\text{vec}(\mathbf{A}') = \text{vec}(\mathbf{A})$ for any matrix $\mathbf{A} \in \mathcal{R}^{p\times p}$. Thus $\hat{\mathbf{A}}_1 = (\hat{\mathbf{W}}' \otimes \mathbf{I}_p)\mathbf{K} + \mathbf{I}_{p^2} \to_{\mathbb{P}} \mathbf{K} + \mathbf{I}_{p^2} = \mathbf{A}_1$.

Let now $\hat{\mathbf{Y}} = \sum_{ab}\hat{\mathbf{W}}'\hat{\mathbf{C}}^{ab}\hat{\mathbf{W}}\text{diag}(\hat{\mathbf{D}}^{ab})$. Eq. (32) gives that $\sqrt{n}\hat{\mathbf{Y}}$ is asymptotically symmetric. That is, $\sqrt{n}\hat{\mathbf{Y}} = \sqrt{n}\hat{\mathbf{Y}}' + o_p(1)$. By applying $\hat{\mathbf{C}}^{ab} = \hat{\mathbf{C}}^{ab} - \delta_{ab}\kappa_a\mathbf{E}^{aa} + \delta_{ab}\kappa_a\mathbf{E}^{aa}$ and Slutsky's theorem, we obtain

$$\begin{aligned}\sqrt{n}\hat{\mathbf{Y}} &= \sum_{ab}\hat{\mathbf{W}}'\sqrt{n}(\hat{\mathbf{C}}^{ab} - \delta_{ab}\kappa_a\mathbf{E}^{aa})\hat{\mathbf{W}}\text{diag}(\hat{\mathbf{D}}^{ab}) + \sum_{ab}\hat{\mathbf{W}}'\sqrt{n}\delta_{ab}\kappa_a\mathbf{E}^{aa}\hat{\mathbf{W}}\text{diag}(\hat{\mathbf{D}}^{ab})\\ &= \sum_{a}\kappa_a\sqrt{n}(\hat{\mathbf{C}}^{aa} - \kappa_a\mathbf{E}^{aa})\mathbf{E}^{aa} + \sum_{a}\sqrt{n}\kappa_a\hat{\mathbf{W}}'\mathbf{E}^{aa}\hat{\mathbf{W}}\text{diag}(\hat{\mathbf{D}}^{aa}) + o_p(1).\end{aligned}$$

Let $\sqrt{n}\hat{\mathbf{F}}_1 = \sum_a\kappa_a\sqrt{n}(\hat{\mathbf{C}}^{aa} - \kappa_a\mathbf{E}^{aa})\mathbf{E}^{aa}$. Applying $\hat{\mathbf{D}}^{aa} = \hat{\mathbf{W}}'\hat{\mathbf{C}}^{aa}\hat{\mathbf{W}} = \hat{\mathbf{W}}'(\hat{\mathbf{C}}^{aa} - \kappa_a\mathbf{E}^{aa})\hat{\mathbf{W}} + \kappa_a\hat{\mathbf{W}}'\mathbf{E}^{aa}\hat{\mathbf{W}}$, we obtain

$$\begin{aligned}\sqrt{n}\hat{\mathbf{Y}} &= \sum_{a}\kappa_a\hat{\mathbf{W}}'\mathbf{E}^{aa}\hat{\mathbf{W}}\text{diag}\left(\hat{\mathbf{W}}'\sqrt{n}(\hat{\mathbf{C}}^{aa} - \kappa_a\mathbf{E}^{aa})\hat{\mathbf{W}}\right)\\ &\quad + \sqrt{n}\sum_{a}\kappa_a\hat{\mathbf{W}}'\mathbf{E}^{aa}\hat{\mathbf{W}}\text{diag}\left(\kappa_a\hat{\mathbf{W}}'\mathbf{E}^{aa}\hat{\mathbf{W}}\right) + \sqrt{n}\hat{\mathbf{F}}_1 + o_p(1),\\ &= \sum_{a}\kappa_a\mathbf{E}^{aa}\text{diag}\left(\sqrt{n}(\hat{\mathbf{C}}^{aa} - \kappa_a\mathbf{E}^{aa})\right)\\ &\quad + \sqrt{n}\sum_{a}\kappa_a^2\hat{\mathbf{W}}'\mathbf{E}^{aa}\hat{\mathbf{W}}\text{diag}\left(\hat{\mathbf{W}}'\mathbf{E}^{aa}\hat{\mathbf{W}}\right) + \sqrt{n}\hat{\mathbf{F}}_1 + o_p(1)\end{aligned}$$

Let $\sqrt{n}\hat{\mathbf{F}}_2 = \sum_a\kappa_a\mathbf{E}^{aa}\text{diag}(\sqrt{n}(\hat{\mathbf{C}}^{aa} - \kappa_a\mathbf{E}^{aa}))$. By plugging in $\hat{\mathbf{W}} = \hat{\mathbf{W}} - \mathbf{I}_p + \mathbf{I}_p$, we obtain

$$\begin{aligned}\sqrt{n}\hat{\mathbf{Y}} &= \sqrt{n}\sum_{a}\kappa_a^2\hat{\mathbf{W}}'\mathbf{E}^{aa}\hat{\mathbf{W}}\text{diag}\left(\hat{\mathbf{W}}'\mathbf{E}^{aa}\hat{\mathbf{W}}\right) + \sqrt{n}\hat{\mathbf{F}}_1 + \sqrt{n}\hat{\mathbf{F}}_2 + o_p(1)\\ &= \sum_{a}\kappa_a^2\sqrt{n}(\hat{\mathbf{W}}' - \mathbf{I}_p)\mathbf{E}^{aa}\hat{\mathbf{W}}\text{diag}\left(\hat{\mathbf{W}}'\mathbf{E}^{aa}\hat{\mathbf{W}}\right)\\ &\quad + \sum_{a}\kappa_a^2\mathbf{E}^{aa}\sqrt{n}(\hat{\mathbf{W}} - \mathbf{I}_p)\text{diag}\left(\hat{\mathbf{W}}'\mathbf{E}^{aa}\hat{\mathbf{W}}\right)\\ &\quad + \sqrt{n}\sum_{a}\kappa_a^2\mathbf{E}^{aa}\text{diag}\left(\hat{\mathbf{W}}'\mathbf{E}^{aa}\hat{\mathbf{W}}\right) + \sqrt{n}\hat{\mathbf{F}}_1 + \sqrt{n}\hat{\mathbf{F}}_2 + o_p(1).\end{aligned}$$

The terms $\sqrt{n}\sum_a\kappa_a^2\mathbf{E}^{aa}\text{diag}(\hat{\mathbf{W}}'\mathbf{E}^{aa}\hat{\mathbf{W}})$ and $\sqrt{n}\hat{\mathbf{F}}_2$ are symmetric and cancel themselves out in the symmetry identity $\sqrt{n}\hat{\mathbf{Y}} = \sqrt{n}\hat{\mathbf{Y}}' + o_p(1)$, which can also be written as $(\mathbf{I}_{p^2} - \mathbf{K})\sqrt{n}\text{vec}(\hat{\mathbf{Y}}) = o_p(1)$. Let $\hat{\mathbf{G}}^{aa} = \text{diag}(\hat{\mathbf{W}}'\mathbf{E}^{aa}\hat{\mathbf{W}}) \to_{\mathbb{P}} \mathbf{E}^{aa}$ and vectorize to obtain

$$\begin{aligned}&\left(\mathbf{I}_{p^2} - \mathbf{K}\right)\left(\sum_{a}\kappa_a^2[\hat{\mathbf{G}}^{aa}\hat{\mathbf{W}}'\mathbf{E}^{aa}\otimes\mathbf{I}_p]\mathbf{K} + \sum_{a}\kappa_a^2[\hat{\mathbf{G}}^{aa}\otimes\mathbf{E}^{aa}]\right)\sqrt{n}\text{vec}(\hat{\mathbf{W}} - \mathbf{I}_p)\\ &= -\left(\mathbf{I}_{p^2} - \mathbf{K}\right)\sqrt{n}\text{vec}(\hat{\mathbf{F}}_1) + o_p(1).\end{aligned}$$

Thus the matrix $\hat{\mathbf{A}}_2$ in Eq. (34) can be given as

$$\begin{aligned}\hat{\mathbf{A}}_2 &= \left(\mathbf{I}_{p^2} - \mathbf{K}\right)\left(\sum_a \kappa_a^2[\hat{\mathbf{G}}^{aa}\hat{\mathbf{W}}'\mathbf{E}^{aa} \otimes \mathbf{I}_p]\mathbf{K} + \sum_a \kappa_a^2[\hat{\mathbf{G}}^{aa} \otimes \mathbf{E}^{aa}]\right)\\ &\rightarrow_{\mathbb{P}} \left(\mathbf{I}_{p^2} - \mathbf{K}\right)\left(\sum_a \kappa_a^2[\mathbf{E}^{aa} \otimes \mathbf{I}_p]\mathbf{K} + \sum_a \kappa_a^2[\mathbf{E}^{aa} \otimes \mathbf{E}^{aa}]\right)\\ &= \left(\mathbf{I}_{p^2} - \mathbf{K}\right)\sum_a \kappa_a^2[\mathbf{E}^{aa} \otimes \mathbf{I}_p]\mathbf{K}\\ &= \sum_a \kappa_a^2[\mathbf{E}^{aa} \otimes \mathbf{I}_p]\mathbf{K} - \sum_a \kappa_a^2[\mathbf{I}_p \otimes \mathbf{E}^{aa}]\\ &= [\mathbf{D} \otimes \mathbf{I}_p]\mathbf{K} - [\mathbf{I}_p \otimes \mathbf{D}] = \mathbf{A}_2,\end{aligned}$$

where we have used the identities $(\mathbf{I}_{p^2} - \mathbf{K})\sum_a \kappa_a^2[\mathbf{E}^{aa} \otimes \mathbf{E}^{aa}] = \mathbf{0}$ and $\mathbf{K}[\mathbf{E}^{aa} \otimes \mathbf{I}_p]\mathbf{K} = [\mathbf{I}_p \otimes \mathbf{E}^{aa}]$ and the notation $\mathbf{D} = \sum_a \kappa_a^2 \mathbf{E}^{aa}$.

Taking the sum over the two expanded estimating equations in Eq. (34), we now obtain,

$$(\hat{\mathbf{A}}_1 + \hat{\mathbf{A}}_2)\sqrt{n}\mathrm{vec}(\hat{\mathbf{W}} - \mathbf{I}_p) = -\left(\mathbf{I}_{p^2} - \mathbf{K}\right)\sqrt{n}\mathrm{vec}(\hat{\mathbf{F}}_1) + o_p(1), \tag{35}$$

where $\hat{\mathbf{A}}_1 + \hat{\mathbf{A}}_2 \rightarrow_{\mathbb{P}} \mathbf{A} = \mathbf{A}_1 + \mathbf{A}_2 = [\mathbf{D} \otimes \mathbf{I}_p]\mathbf{K} - [\mathbf{I}_p \otimes \mathbf{D}] + \mathbf{K} + \mathbf{I}_{p^2}$. The right-hand side of Eq. (35) equals

$$\left(\mathbf{K} - \mathbf{I}_{p^2}\right)\sqrt{n}\mathrm{vec}(\hat{\mathbf{F}}_1) + o_p(1) = \sqrt{n}\mathrm{vec}(\hat{\mathbf{F}}_1{}' - \hat{\mathbf{F}}_1) + o_p(1), \tag{36}$$

which is $O_p(1)$ as $\sqrt{n}\hat{\mathbf{F}}_1 = O_p(1)$. To see that $\mathbf{A}$ is invertible, we compute its determinant and verify that it is non-zero under Assumption 1. Recall that Assumption 1 says that there is maximally one zero diagonal element in $\mathbf{D}$.

Observe first that in the case $p = 2$, the matrix $\mathbf{A}$ takes the form,

$$\begin{pmatrix} 2 & 0 & 0 & 0\\ 0 & 1-\kappa_2^2 & 1+\kappa_1^2 & 0\\ 0 & 1+\kappa_2^2 & 1-\kappa_1^2 & 0\\ 0 & 0 & 0 & 2 \end{pmatrix},$$

where the 2×2 diagonal block has determinant equal to $-2(\kappa_1^2 + \kappa_2^2)$. As the determinant of a block diagonal matrix is the product of the determinants of the blocks, we have that $\det(\mathbf{A}) = -8(\kappa_1^2 + \kappa_2^2)$, verifying our claim in the case $p = 2$.

For general p, the matrix $\mathbf{A}$ can, after a suitable permutation of its rows and columns, not affecting the value of the determinant, be seen to consist of similar blocks as in the case $p = 2$. For each diagonal element of $\sqrt{n}(\hat{\mathbf{W}} - \mathbf{I}_p)$ (the elements $a + (a-1)p$, $a \in \{1, \ldots, p\}$, of $\sqrt{n}\mathrm{vec}(\hat{\mathbf{W}} - \mathbf{I}_p)$), we get a 1×1 diagonal block equal to 2. For each (a, b)th off-diagonal element in the lower triangle of $\mathbf{A}$, we get a 2×2 diagonal block,

$$\begin{pmatrix} 1-\kappa_b^2 & 1+\kappa_a^2\\ 1+\kappa_b^2 & 1-\kappa_a^2 \end{pmatrix}, \tag{37}$$

with the determinant equal to $-2(\kappa_a^2 + \kappa_b^2)$. As the number of diagonal elements is p and the number of off-diagonal

elements in the lower triangle is $p(p-1)/2$, the total determinant is,

$$\det(\mathbf{A}) = 2^p(-2)^{p(p-1)/2}\prod_{a>b}(\kappa_a^2+\kappa_b^2),$$

which is non-zero if and only if Assumption 1 holds.

Multiply now both sides of Eq. (35) by $(\hat{\mathbf{A}}_1+\hat{\mathbf{A}}_2)^{-1}$ (which is asymptotically well-defined as $\det(\mathbf{A}) \neq 0$) and invoke Slutsky's theorem to obtain,

$$\sqrt{n}\mathrm{vec}(\hat{\mathbf{W}}-\mathbf{I}_p) = (\hat{\mathbf{A}}_1+\hat{\mathbf{A}}_2)^{-1}\sqrt{n}\mathrm{vec}(\hat{\mathbf{F}}_1{}'-\hat{\mathbf{F}}_1) + o_p(1),$$

where the right-hand side has the same limiting distribution as $\mathbf{A}^{-1}\sqrt{n}\mathrm{vec}(\hat{\mathbf{F}}_1{}'-\hat{\mathbf{F}}_1)$, i.e., a multivariate normal distribution. This reveals that $\sqrt{n}(\hat{\mathbf{W}}-\mathbf{I}_p)$ is indeed $O_p(1)$. To obtain asymptotic expressions for the elements of $\sqrt{n}(\hat{\mathbf{W}}-\mathbf{I}_p)$, we inspect its diagonal and off-diagonal elements separately.

Each element of $\sqrt{n}\mathrm{vec}(\hat{\mathbf{W}}-\mathbf{I}_p)$ corresponding to a diagonal element of $\sqrt{n}(\hat{\mathbf{W}}-\mathbf{I}_p)$ is associated with a 1×1 diagonal block in $\mathbf{A}^{-1}$ equal to $1/2$. This block picks the corresponding diagonal element from the right-hand side of Eq. (36) into the expression of $\sqrt{n}(\hat{w}_{aa}-1)$. However, the diagonal elements of the expression inside the vectorization operator in Eq. (36) are clearly zero and we have,

$$\sqrt{n}(\hat{w}_{aa}-1) = o_p(1), \quad \forall a \in \{1,\ldots,p\}. \tag{38}$$

Take now a pair of off-diagonal elements (a,b), (b,a), with $a>b$. Each such pair has a 2×2 matrix of the form of Eq. (37) associated with it, with the upper row corresponding to the element (a,b). By Cramer's rule, the inverse of the matrix is

$$\frac{1}{2(\kappa_a^2+\kappa_b^2)}\begin{pmatrix}\kappa_a^2-1 & \kappa_a^2+1\\ \kappa_b^2+1 & \kappa_b^2-1\end{pmatrix}.$$

The corresponding rows in $\mathbf{A}^{-1}\sqrt{n}\mathrm{vec}(\hat{\mathbf{F}}_1{}'-\hat{\mathbf{F}}_1)$ can be given as

$$\frac{1}{2(\kappa_a^2+\kappa_b^2)}\begin{pmatrix}\kappa_a^2-1 & \kappa_a^2+1\\ \kappa_b^2+1 & \kappa_b^2-1\end{pmatrix}\begin{pmatrix}[\hat{\mathbf{F}}_1{}'-\hat{\mathbf{F}}_1]_{ab}\\ [\hat{\mathbf{F}}_1{}'-\hat{\mathbf{F}}_1]_{ba}\end{pmatrix} = \frac{1}{(\kappa_a^2+\kappa_b^2)}\begin{pmatrix}-[\hat{\mathbf{F}}_1{}'-\hat{\mathbf{F}}_1]_{ab}\\ [\hat{\mathbf{F}}_1{}'-\hat{\mathbf{F}}_1]_{ab}\end{pmatrix},$$

where $[\mathbf{M}]_{ab}$ denotes the (a,b) element of a matrix $\mathbf{M}$ and we have used $[\hat{\mathbf{F}}_1{}'-\hat{\mathbf{F}}_1]_{ba} = -[\hat{\mathbf{F}}_1{}'-\hat{\mathbf{F}}_1]_{ab}$ to simplify the expression. As $[\hat{\mathbf{F}}_1{}'-\hat{\mathbf{F}}_1]_{ab} = \kappa_a\sqrt{n}\hat{\mathbf{C}}_{ba}^{aa} - \kappa_b\sqrt{n}\hat{\mathbf{C}}_{ab}^{bb} = \kappa_a\sqrt{n}\hat{\mathbf{C}}_{ab}^{aa} - \kappa_b\sqrt{n}\hat{\mathbf{C}}_{ba}^{bb}$, we obtain

$$\sqrt{n}\hat{w}_{ab} = \frac{\kappa_a\sqrt{n}\hat{\mathbf{C}}_{ab}^{aa} - \kappa_b\sqrt{n}\hat{\mathbf{C}}_{ba}^{bb}}{\kappa_a^2+\kappa_b^2} + o_p(1) \tag{39}$$

for all $a \neq b$, $a,b \in \{1,\ldots,p\}$. The expressions in Eqs. (38) and (39) now match exactly to those obtained in the proof of (Virta et al., 2018, Theorem 2) (with a different $o_p(1)$-sequence, though) and the limiting variances can be derived as in (Virta et al., 2018, Corollary 1).

references

Bonhomme, S. and Robin, J.-M. (2009) Consistent noisy independent component analysis. *J. Econometrics*, **149**, 12–25.

Eaton, M. L. and Tyler, D. E. (1991) On Wielandt's inequality and its application to the asymptotic distribution of the eigenvalues of a random symmetric matrix. *Ann. Statist.*, 260–271.

Miettinen, J., Illner, K., Nordhausen, K., Oja, H., Taskinen, S. and Theis, F. J. (2016) Separation of uncorrelated stationary time series using autocovariance matrices. *J. Time Series Anal.*, **37**, 337–354.

Van der Vaart, A. W. (1998) *Asymptotic statistics*. Cambridge University Press.

Virta, J., Li, B., Nordhausen, K. and Oja, H. (2017) Independent component analysis for tensor-valued data. *J. Multivariate Anal.*, **162**, 172–192.

— (2018) JADE for tensor-valued observations. *J. Comput. Graph. Statist.*, **27**, 628–637.
